# Can Digital PCR Enhance the Detection of *Legionella pneumophila* in Legionnaires’ Disease Cases?

**DOI:** 10.3390/pathogens15090989

**Published:** 2026-09-17

**Authors:** Fabiola Mancini, Enza Bruna Bonazza, Antonino Bella, Antonietta Girolamo, Massimo Mentasti, Maria Luisa Ricci, Maria Scaturro

**Affiliations:** 1 Department of Infectious Diseases, Istituto Superiore di Sanità, 00161 Rome, Italy; 2 Public Health Wales NHS Trust, Cardiff CF10 4BZ, UK; 3 ESCMID Study Group for Legionella Infections (ESGLI), 4051 Basel, Switzerland

**Keywords:** *Legionella pneumophila*, Legionnaires’ disease, digital PCR, qPCR, UAT, culture, diagnosis

## Abstract

The laboratory diagnosis of Legionnaires’ disease (LD) remains a significant challenge due to the limitations of current methods. Most LD cases are diagnosed through the detection of *Legionella* urine antigen using commercial assays that are usually specific just for *Legionella pneumophila* (Lp) serogroup 1. Isolating Lp through culture is rarely done and difficult to achieve, especially due to two important issues: the low sensitivity of the method and the fact that it is difficult to obtain respiratory secretions from a significant proportion of patients. Real-time PCR (qPCR) has significantly improved LD diagnosis, indicating that molecular methods based on *Legionella* DNA detection can be highly effective. This study aimed to adapt a qPCR assay to a digital PCR (dPCR) assay, taking advantage of the more sensitive and more sophisticated dPCR technology. In particular, the study focused on 42 respiratory specimens, including bronchoalveolar lavage, tracheal aspirates, and sputum samples, with a particular focus on culture-negative specimens presumed to contain low concentrations of *Legionella* DNA, which were considered particularly suitable for assessing the potential of dPCR to improve the diagnosis of Legionnaires’ disease. The results of culture, qPCR, and dPCR of respiratory samples were compared with each other and with data from urinary antigen tests (UATs) available for the corresponding patients. Overall, dPCR detected *Legionella* DNA in additional respiratory specimens compared with qPCR, corresponding to a 19% increase in the number of qPCR-negative specimens yielding a positive dPCR result. dPCR also detected *Legionella* DNA in specimens from patients with negative urinary antigen results. These findings suggest that dPCR may have potential as a complementary molecular approach for improving *Legionella* DNA detection in respiratory specimens, particularly in culture-negative samples. However, the clinical significance of dPCR-positive/qPCR-negative results could not be independently confirmed in this study and requires further investigation.

## 1. Introduction

Digital PCR (dPCR), a third-generation PCR method, has emerged as a promising tool for the rapid and precise diagnosis of infectious diseases caused by various pathogens, such as bacteria, viruses and protozoa; it offers significant advantages over traditional DNA-based methods like block-based PCR and real-time PCR [1].

By randomly distributing DNA molecules into many partitions and creating independent sub-reactions where target sequences are efficiently concentrated within isolated microreactors, dPCR enables the absolute quantification of target nucleic acids present in a reaction. This technique is based on end-point PCR techniques and Poisson statistics, eliminating the need for calibration curves. It improves accuracy for low-abundance targets and may also allow for a higher tolerance to inhibitors present in a sample [2,3]. Among the dPCR platforms, some utilize droplet digital PCR technology to partition PCR reactions into approximately 20,000 nanoliter-scale droplets suspended in oil, which are individually analyzed for fluorescence after thermal cycling [4].

Thanks to features such as high sensitivity, high throughput, and robust quantification, digital PCR has emerged as a powerful tool for molecular diagnostics. It has been used to detect bloodstream infections in intensive care units, identify antimicrobial resistance genes, and diagnose parasitic and viral infections [5,6,7,8]. dPCR assays have been developed to detect monkeypox and papilloma virus infections [9,10], outliers from a time series of pathogen concentrations for wastewater-based pathogen surveillance [11], and to diagnose *Helicobacter pylori*, brucellosis and several other pathogens [12,13].

LD is a severe form of pneumonia predominantly caused by *Legionella pneumophila* (Lp), although other *Legionella* species may also be involved. It can entail admission to the intensive care unit in almost one-third of cases, and among these cases the mortality rate ranges from 4% to 40% [14].

According to annual reports from the European Centre for Disease Prevention and Control (ECDC), the notification rate for LD has been constantly increasing (https://www.ecdc.europa.eu/sites/default/files/documents/LEGI_AER_2023_Report.pdf accessed on 26 May 2026), especially after the introduction of a diagnostic test based on the detection of *Legionella* urinary antigen (UAT). In 2024, the notification rates in Europe and Italy were 3.4 and 7.8 cases per 100,000 inhabitants (https://atlas.ecdc.europa.eu/public/ accessed on 26 May 2026) [15,16], respectively. A similar trend is observed worldwide (https://www.cdc.gov/legionella/ accessed on 26 May 2026). The UAT is the primary diagnostic method for LD, as 79% of cases were reported as diagnosed using this method, while only 9.7% and 12.7% of cases are diagnosed by culture and PCR methods, respectively [15]. Commercially available UATs are primarily designed to detect *Legionella pneumophila* serogroup 1 (Lp1), while only a limited number of assays are capable of identifying other Lp serogroups or few *Legionella* species, leaving many non-Lp1 and other *Legionella* species infections undetected and under-reported [17,18,19].

PCR can enhance the identification of *Legionella pneumophila* serogroups 1–15 (Lp1–15) as well as non-*pneumophila* species [20,21,22]. In 2015, a multiplex qPCR assay was validated for the accurate detection of Lp, allowing for differentiation between serogroup 1 and non-serogroup 1 [23].

According to the European Union case definition for Legionnaires’ disease (https://eur-lex.europa.eu/legal-content/EN/TXT/PDF/?uri=CELEX:32018D0945&from=EN#page=26 accessed on 26 May 2026), methods based on “Detection of Legionella spp. nucleic acid in respiratory secretions, lung tissue or any normally sterile” still identify probable cases. However, real-time PCR has increased the detection of LD cases by 18% to 30% [20,21,22]. For this reason, in 2020, the CDC updated the LD case definition, changing nucleic acid amplification testing (NAAT)-positive cases from the suspect to the confirmed category (https://www.cdc.gov/investigate-legionella/php/data-research/case-definitions.html, accessed on 26 May 2026).

In this study, we explored the performance of digital PCR (dPCR) for the detection of *Legionella pneumophila* compared to current methods. Culture is the gold standard for LD diagnosis; however its limitations are well known [14]. For the purpose of this study, DNA extracts presumed to contain very low numbers of Lp genomic copies, because they were negative by culture, were analyzed. The performance of dPCR was assessed through comparison with real-time PCR (qPCR) results, as well as with culture and urinary antigen test (UAT) data available for the corresponding samples.

## 2. Materials and Methods

### 2.1. Sample Selection and Inclusion Criteria

Over the past three years, the Italian National Reference Laboratory for *Legionella* has received a variable number of respiratory samples (ranging from 22 to 192 per year) for the diagnosis and confirmatory testing of LD, which is routinely performed using culture and qPCR. As the primary objective of this study was to investigate the potential of dPCR to improve the detection of Lp, culture-negative respiratory samples were preferentially selected as well as samples that showed a late fluorescence signal by qPCR. A total of 42 respiratory specimens, including bronchoalveolar lavage, tracheal aspirates, and sputum samples, were chosen based on culture results: 25 (59.5%) were culture-negative, and 17 (40.5%) were culture-positive. UAT results were available for all 42 samples and were included in the comparative analysis.

### 2.2. Sample Processing and DNA Extraction

For cultural examination, respiratory secretions were first treated with Sputasol (Thermo Fisher Scientific, Waltham, MA, USA), according to the manufacturer’s instructions, to liquefy the samples. For each liquefied sample, three 100 μL aliquots were plated on BCYE (Thermo Fisher Scientific, Waltham, MA, USA) and GVPC (Thermo Fisher Scientific, Waltham, MA, USA) agar plates: one aliquot was plated without any treatment, the second after being treated at 50 °C for 30 min, and the third after being treated with a 1:10 diluted acid solution (pH 2.0). All culture plates were then incubated at 36 ± 1 °C for at least 10 days. Suspected colonies were then sub-cultured on BCYE without cysteine, and if no growth was observed, they were then tested using the Legionella latex test (Thermo Fisher Scientific, Waltham, MA, USA), a rapid agglutination assay for the identification of *Legionella*.

DNA extraction was performed on 200 μL of respiratory samples using the QiaCube (Qiagen, Hilden, Germany) extraction platform and the DNeasy Blood and Tissue Kit (Qiagen, Hilden Germany). An extraction control was included in each DNA extraction procedure to monitor the extraction process and identify potential extraction failures or contamination. After extraction, the DNA samples were stored at −20 °C until further use.

### 2.3. qPCR and dPCR

Applied Biosystems QuantStudio Absolute Q Digital PCR System (Thermo Fisher Scientific, Waltham, MA, USA) and the Rotor-Gene thermal cycler (Qiagen, Hilden, Germany) were used for all dPCR and qPCR experiments, respectively.

Multiplex qPCR, targeting *mip* (detecting Lp1-15) and *wzm* (specifically detecting Lp1) and the internal positive control green fluorescent protein, *gfp*, genes, as already described [23], was used to analyze all DNA extracts. Reactions were performed using the Rotor-Gene Q (Qiagen, Hilden, Germany) in a final volume of 20 μL, including 10 μL of 2× QuantiNova Multiplex PCR Kit (Qiagen, Hilden, Germany) and 5 μL of DNA extract. The thermal protocol was performed, as previously described [23]. Target quantification expressed as Genomic Unit (GU)/μL was obtained using a standard curve generated by testing 10-fold serial dilutions of a *L. pneumophila* sg1 DNA quantification standard (Minerva Biolabs, Berlin, Germany). Negative and positive controls were also added on each run. The results were analyzed in the green, yellow and red channels of Rotor-Gene Q software v2.1.0.9, targeting *mip, wzm* and *gfp*, respectively. The Rotor-Gene Q software was set to ignore fluorescent signals of the first 15 cycles and to use 0.025 as a threshold value.

The same primers and probes used for the qPCR assay were also used for dPCR assays in combination with Applied Biosystems QuantStudio Absolute Q Digital PCR System (Thermo Fisher Scientific, Waltham, MA, USA); however, in this case, primers and probes of the *gfp* internal control assay were excluded as reaction partitioning provides dPCR with increased tolerance to inhibiting substances; therefore an internal control is not required [24]. Following the manufacturer’s instructions, dPCR reaction mixes were prepared using 1.8 μL of Absolute Q™ DNA Digital PCR Master Mix (5×), 5 μL of DNA template, 0.45 μL of each primer (20×) (final concentration, 900 nM) plus TaqMan Probes (final concentration, 250 nM) in a final volume of 9 μL. The amplification protocol was performed with an initial denaturation at 96 °C for 10 min, followed by 40 cycles of denaturation at 96 °C for 5 s, and annealing-extension at 60 °C for 15 s. Each run included negative control. The results were analyzed in green (*wzm*) and yellow (*mip*) channels using the QuantiStudio Absolute Q Digital PCR System software Version 6.3; the number of GU/μL was also determined. Duplicate independent runs of both qPCR and dPCR were carried out for all the DNA extracts.

### 2.4. qPCR and Digital PCR: Standard Curve and Limit of Detection (LoD)

The limit of detection (LoD) was defined as the lowest concentration of DNA standard at which at least 90% of replicates were positive. A Lp1 commercial standard DNA was employed to confirm the efficiency and LoD of the qPCR, as previously described [23]. In order to confirm the linearity of the method and for the standard curve production, 10-fold serial dilutions of the standard were prepared to obtain 50,000, 5000, 500, 50 and 5 Genomic Units (GU)/reaction and tested in triplicate over 5 different runs. In each run, 5 μL of standard DNA dilutions was tested together with a negative control.

Primers and probes of the qPCR assay were also used for the dPCR assay with the Applied Biosystems QuantStudio Absolute Q Digital PCR System, following the manufacturer’s protocol. The LoD of the dPCR was determined through five runs, where ten replicates of 50, 5, 2 GU/reaction of the DNA standards and negative control were tested. To calculate the LoD, the green (*wzm*) and yellow (*mip*) channels were selected.

### 2.5. Statistical Analysis

Data obtained from the 42 clinical samples were all collected in an Excel file and then transferred onto Stata software (version 11.2, Stata Corp, College Station, TX, USA) for statistical analysis. For statistical analysis, although culture is traditionally considered the reference method for LD diagnosis, qPCR was also selected as comparator due to its higher analytical sensitivity and its established role in molecular diagnosis of LD.

The concordance was evaluated using Cohen’s Kappa coefficient for which K < 0.20 = “poor,” K = 0.20–0.40 = “fair,” K = 0.40–0.60 = “moderate,” K = 0.60–0.80 = “good,” and K = 0.80–1.00 = “very good” [24]. Specificity and sensitivity, as well as positive and negative predictive values (PPV and NPV, respectively), and 95% confidence intervals (CI) of dPCR vs. qPCR and culture were also calculated.

## 3. Results

### 3.1. Culture and UAT

As shown in Table 1, 19 out of the 42 clinical samples were negative by both culture and UAT, while 12 were positive with both methods. Five samples were positive only by culture, and six were only positive by UAT.

The majority (14 out of 17) of culture-positive samples were Lp1, while Lp sg2-15 was cultured from the remaining three samples which had a corresponding negative UAT result.

### 3.2. dPCR vs. qPCR

The LoD of dPCR was calculated to be 0.23 GU/μL which corresponds to approximately 2 GU per reaction.

The comparison between dPCR and qPCR is detailed in Table 2 and in Table S1. The agreement between dPCR and qPCR was noted in 33/42 (78.57%) samples (Kappa 0.36, *p* = 0.007). As reported in Table 3, the sensitivity of dPCR compared to qPCR was 96.67%, while the specificity was 33%, due to eight samples found positive in dPCR and negative in qPCR. The negative predictive value and the positive predictive value were 80% (CI 95%: 67.90–92.10%) and 78.38% (CI 95%: 65.93–90.83%), respectively. In Supplementary Material, Table S1, genomic copies for both qPCR and dPCR calculated for both *wzm* and *mip* gene targets are reported. In Supplementary Material, Figure S1, a graphical comparison of the copy number estimates obtained by the two methods for four discordant samples, which were positive only in dPCR (ID 9, 23, 28 and 34 in bold in Table S1), is shown. Additionally, for these discordant samples, 2D graphs and the qPCR graphical amplification are shown in Figures S2 and S3, respectively.

### 3.3. dPCR vs. Culture

As the isolation by culture is the gold standard for LD diagnosis and, according to the case definition, it defines a confirmed case, the results obtained by dPCR were also compared with those obtained by culture. In Table 4, the number of positive and negative samples is reported for both methods, while in Table 5 the specificity, sensitivity, positive predictive and negative predictive values are indicated. The agreement between dPCR and culture was noted in 22/42 (52.4%) samples (K0.17, *p* = 0.0495).

### 3.4. Comparison of All Methods

The results of UAT, culture, qPCR and dPCR are detailed in Table S1. Twelve out of the 42 clinical samples were positive in all the tests performed, and they were all related to LD cases caused by Lp1; on the other hand, only three samples were negative in all the tests. Four out of the 42 samples were positive only with dPCR, while one was positive exclusively with qPCR. Eleven samples were positive only by dPCR and qPCR, all were positive for *mip*, and three were also positive for *wzm*. A further five UAT-positive–culture-negative samples were also positive in dPCR, and all were *mip*-positive and three also *wzm*-positive. The Lp quantification in these samples ranged from 2 to 66 GU/μL.

## 4. Discussion

In this study, an in-house dPCR assay was used to detect Lp in DNA extracted from respiratory samples. The assay was based on primers and probes already designed and validated for detecting Lp (*mip*) and Lp sg1 (*wzm*) markers using qPCR [23].

The results showed high agreement between dPCR and qPCR and a lower specificity (33%) of dPCR compared to qPCR due to eight samples found positive in dPCR but negative in qPCR, which improved the Lp diagnosis by 19% in comparison to qPCR. As is known, specificity is defined as the proportion of true negatives, found by the reference test (in this case the qPCR), with respect to the total negatives plus the false positives. One out of the eight samples was a true positive, being culture-positive, as were two out of the eight that were UAT-positive. Five out of the eight samples were positive in dPCR to the *mip* gene targeting Lp1-15, and two out of the five were from a patient with a UAT-positive result. Indeed, although most commercially available UATs are primarily designed to detect Lp1, cross-reactivity with non-serogroup 1 has been reported for some assays, with variable sensitivity across serogroups [25,26]. Therefore, dPCR-positive/UAT-negative results should not necessarily be interpreted as false-positive dPCR results, particularly when the bacterial burden is low or when infection is caused by a non-serogroup 1 strain. Rather, these discordant results may reflect the broader analytical target range and improved detection capability of dPCR and not true false positives.

Conversely, one specimen was positive by qPCR but negative by dPCR (ID33, Table S1). This specimen showed a low target concentration by qPCR, and the absence of detectable target molecules by dPCR raises the possibility of a low-level qPCR signal or a false-positive result. However, this interpretation cannot be confirmed because no independent reference method was available to resolve this discordance. Therefore, these results should be interpreted cautiously, and further investigation is warranted to determine the clinical significance of discordant qPCR/dPCR results.

Additionally, when culture results were used as the reference method, dPCR showed 100% sensitivity and 100% NPV, which combined are of great diagnostic relevance, as samples tested negative by dPCR are likely to be “true negatives”.

Twenty-five out of 42 samples (59%) were negative by culture, and the corresponding results by dPCR and qPCR were either negative or weak positive (Table S1. Several samples positive by dPCR showed a very low number of positive molecules, and four samples positive only by dPCR were characterized by containing between three and 13.33 positive molecules (Table S1). In the context of dPCR, “positive molecules” refers to the number of individual DNA target copies that are detected as positive amplification events across the thousands of micro-reactions into which the sample is partitioned; each positive partition corresponds to the presence of at least one target DNA molecule [1]. After establishing the dPCR limit of detection (LoD) at two positive events, the detection of three to four positive molecules, clearly distinguishable by fluorescence, supports the robustness of the method. This makes dPCR more reliable than interpreting a positive qPCR result based solely on cycle threshold values for identifying Lp in respiratory samples.

The clinical relevance of increased analytical sensitivity deserves consideration in the context of LD. Detection of low concentrations of *Lp* DNA may be particularly valuable when the bacterial burden in respiratory specimens is low, as these infections may be missed by culture, especially when specimens are collected after antimicrobial treatment. Molecular assays have been reported to be more sensitive than culture for the detection of *Legionella* in lower respiratory tract specimens, and their sensitivity may be less affected by prior antimicrobial therapy [21,27]. Current diagnostic recommendations therefore support the use of molecular testing or culture of lower respiratory tract specimens in combination with urinary antigen testing in order to improve the detection of Legionnaires’ disease and to identify infections caused by *Legionella* species or serogroups not detected by UAT [27,28]. Beyond its diagnostic value, quantification of *Legionella* DNA may provide clinically relevant information on bacterial burden. In a study of patients with legionellosis, the *Legionella* burden measured in lower respiratory tract samples at hospital admission ranged from 1.9 to 8.35 log10 DNA copies/mL, and higher bacterial loads were significantly associated with greater disease severity, the need for intensive care, and prolonged hospitalization [29]. In this context, the ability of dPCR to detect and quantify very low concentrations of Lp DNA could represent an additional advantage over less sensitive qualitative methods. Nevertheless, whether dPCR-derived bacterial load can be reliably used as a prognostic marker requires further investigation in larger clinical cohorts, as detection of bacterial DNA does not necessarily reflect the presence of viable bacteria [28]. Moreover, the present study was not designed to assess associations between bacterial load and clinical outcomes.

Unfortunately, information regarding the stage of the disease at which the samples had been collected and antibiotic therapy was not available. However, it is likely that antibiotic therapy hinders the isolation by culture, causing false negative cultures, while methods based on DNA detection are not affected [20]. dPCR could prove very effective in LD cases where bacteria are difficult to culture due to antibiotic therapy already being administered and/or due to the high burden of contaminating bacterial flora. It could also be advantageous in cases involving immunocompetent individuals, where the immune system is quickly activated to clear the infection.

On the contrary, a limitation of dPCR is that samples with a high bacterial load cannot be accurately quantified, as in sample 31 for which dPCR found over 20,000 positives while qPCR detected 10^7^/10^8^ genomic copies per reaction. High target concentrations approaching the 20,000-nanochamber capacity of the QuantStudio Absolute Q chip led to premature dynamic range saturation; under such conditions, the high probability of multi-molecule occupancy per partition skews the binary readout, thereby inflating Poisson confidence intervals and compromising absolute quantification accuracy. However, this issue can be easily mitigated by testing serial dilutions of the DNA extract [2,30,31,32,33,34].

This study has some limitations that should be considered when interpreting the results. First, the relatively small number of clinical respiratory specimens limits the statistical power of the study and the generalizability of the findings. Second, although the dPCR assay was adapted from an established qPCR assay, an extensive optimization of the dPCR conditions was not performed. Third, the analytical characterization of the assay was limited and did not constitute a complete validation of the dPCR method, including a comprehensive assessment of analytical sensitivity, specificity, linearity, precision, and reproducibility. The present study was primarily designed to provide a preliminary evaluation of the potential of dPCR to improve the detection of L. pneumophila DNA, particularly in respiratory specimens containing low concentrations of target DNA. Therefore, the findings should be considered preliminary, and further studies including larger clinical cohorts and a comprehensive analytical validation of the assay are warranted.

In conclusion, our findings suggest that dPCR may provide improved detection of *L. pneumophila* DNA, particularly in respiratory specimens with low concentrations of target DNA, and may represent a promising complementary molecular approach for the diagnosis of LD. These findings should be considered exploratory and do not, by themselves, support the immediate implementation of dPCR as a routine diagnostic method. Larger clinical studies and comprehensive analytical and clinical validation are warranted to confirm its diagnostic performance and to establish its potential role in routine practice. More broadly, the increasing evidence supporting the diagnostic value of molecular detection of *L. pneumophila* suggests that NAATs may deserve further consideration in future revisions of the European Union Legionnaires’ disease case definition, particularly for cases in which conventional diagnostic methods may have limited sensitivity.

## Figures and Tables

**Table 1:** Number of positive and negative samples by culture and UAT.

		Culture		
		Negative	Positive	Total
**UAT**	**Negative**	19	5	24
	**Positive**	6	12	18
	**Total**	25	17	42

**Table 2:** Number of positive and negative samples found by dPCR and qPCR.

		qPCR		
		Positive	Negative	Total
**dPCR**	**Positive**	29	8	37
	**Negative**	1	4	5
	**Total**	30	12	42

**Table 3:** Sensitivity, specificity, positive predictive value (PPV), and negative predictive value (NPV) of dPCR compared with qPCR.

	Sensitivity	Specificity	PPV	NPV
Result (%)	96.67	33.33	78.38	80.00
95% Conf. Int.	83.3–99.4	13.8–60.9	62.8–88.6	37.6–96.4

**Table 4:** dPCR versus culture.

		Culture		
		Negative	Positive	Total
**dPCR**	**Negative**	5	0	5
	**Positive**	20	17	37
	**Total**	25	17	42

**Table 5:** Sensitivity, specificity, positive predictive value (PPV), and negative predictive value (NPV) of dPCR compared with culture.

	Sensitivity	Specificity	PPV	NPV
Result (%)	100	20%	45.95	100
95% Conf. Int.	100	7.9–32.10	39.87–61.02	100

## Data Availability

The original contributions presented in this study are included in the article/Supplementary Material. Further inquiries can be directed to the corresponding author.

## Supplementary Materials

The following supporting information can be downloaded at: https://www.mdpi.com/article/10.3390/pathogens15090989/s1. Figure S1: Comparison of genomic copy numbers obtained by qPCR and dPCR in discordant samples; Figure S2: Representative 2D dPCR fluorescence plots of low-copy-number discordant samples and corresponding no-template controls (NTCs); Figure S3: Rappresentative qPCR amplification curves of low-copy-number discordant samples Table S1: Comparison of all results collected by UAT, culture, qPCR and dPCR.
