# Supplementary material for: Can Digital PCR Enhance the Detection of Legionella pneumophila in Legionnaires’ Disease Cases?
Source: Pathogens. 2026 Sep 17;15(9):989. doi: 10.3390/pathogens15090989 (PMC13610549; doi:10.3390/pathogens15090989)
Supplement: Supplementary file 1 [file pathogens-15-00989-s001.zip › pathogens-4514914-supplementary.pdf]

Figure S1. Comparison of genomic copy numbers obtained by qPCR and dPCR in discordant samples

A

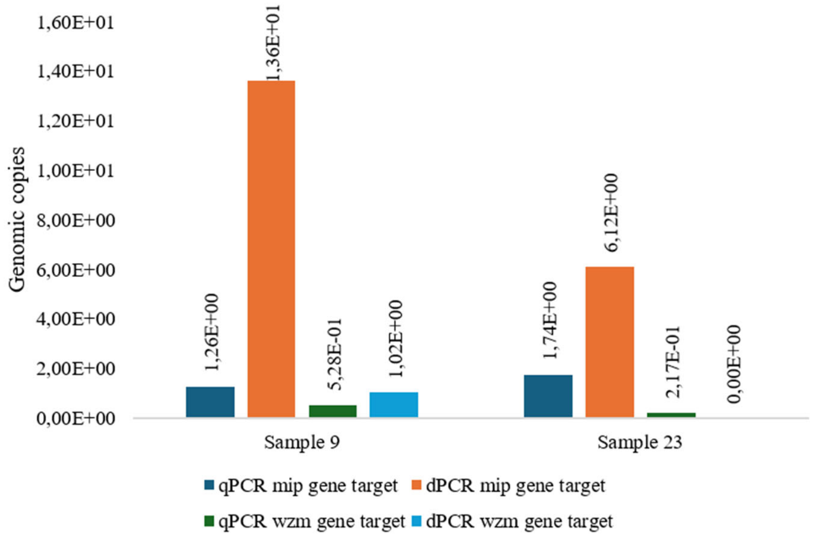

B

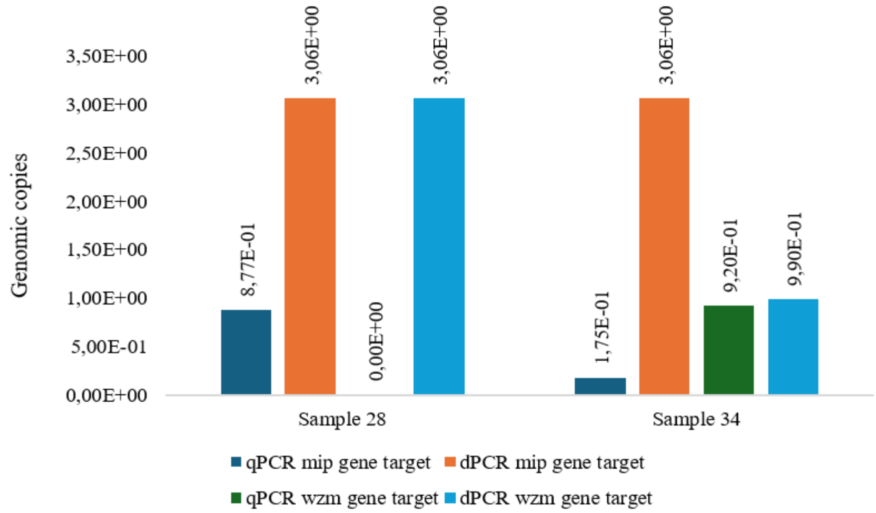

Figure S1. Comparison of genomic copy numbers obtained by qPCR and dPCR for representative discordant samples (ID 9, 23, 28 and 34). Panel A includes samples ID 9 and 23, whereas Panel B includes samples ID 28 and 34. Genomic copy numbers measured for the *mip* and *wzm* gene targets are shown for both methods. All discordant samples exhibited low target concentrations, with dPCR generally providing higher copy number estimates than qPCR, particularly for the *mip* gene target.

Figure S2

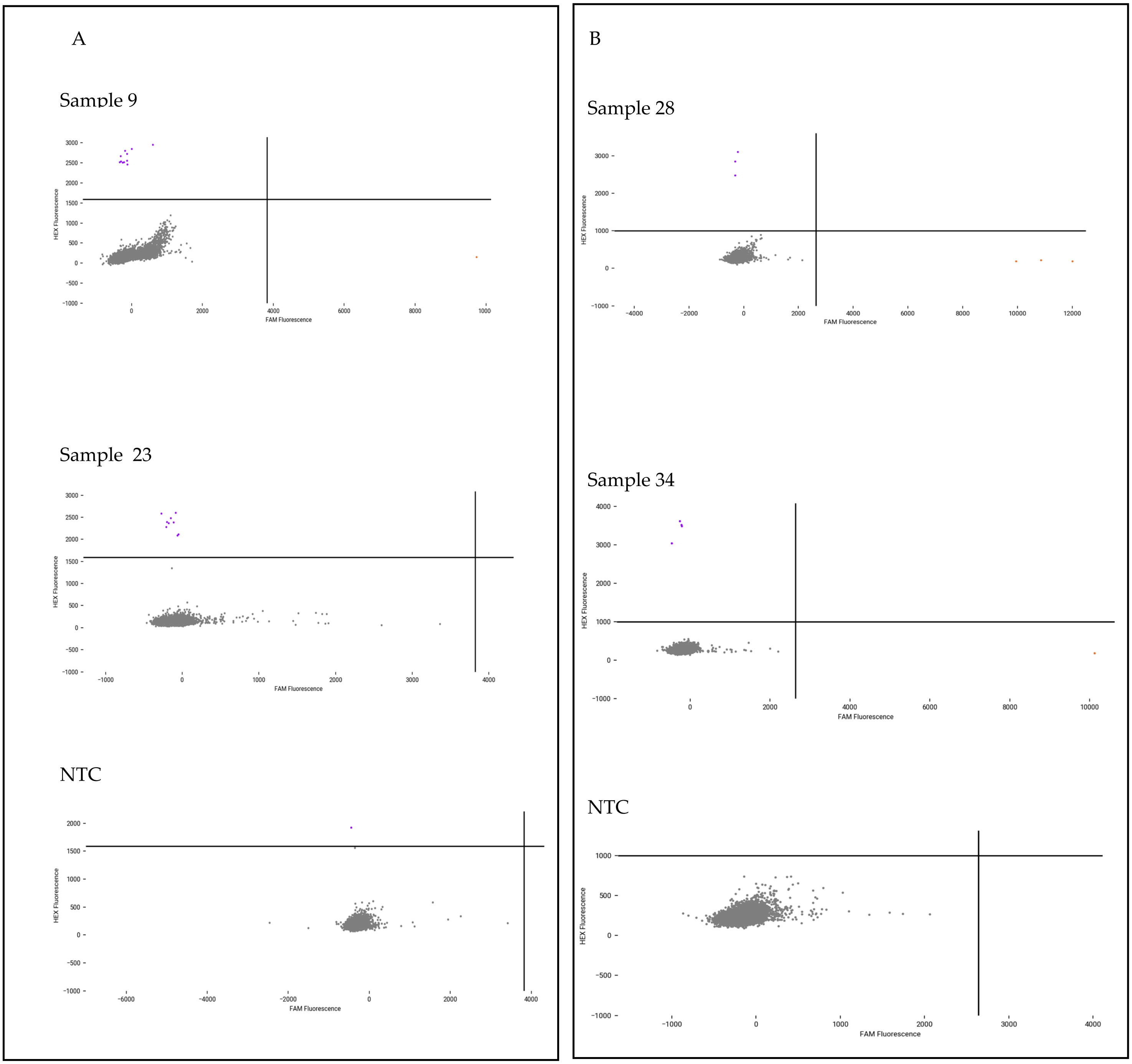

Figure S2. Representative 2D dPCR fluorescence plots of low-copy-number discordant samples and corresponding no-template controls (NTCs). Panel A includes samples ID 9 and ID 23 and the corresponding no-template control (NTC), while Panel B includes samples ID 28 and ID 34 and the corresponding NTC. FAM and HEX fluorescence amplitudes are reported on the x- and y-axes, respectively. Quantitative results for these samples are reported in Table S1.

Figure S3

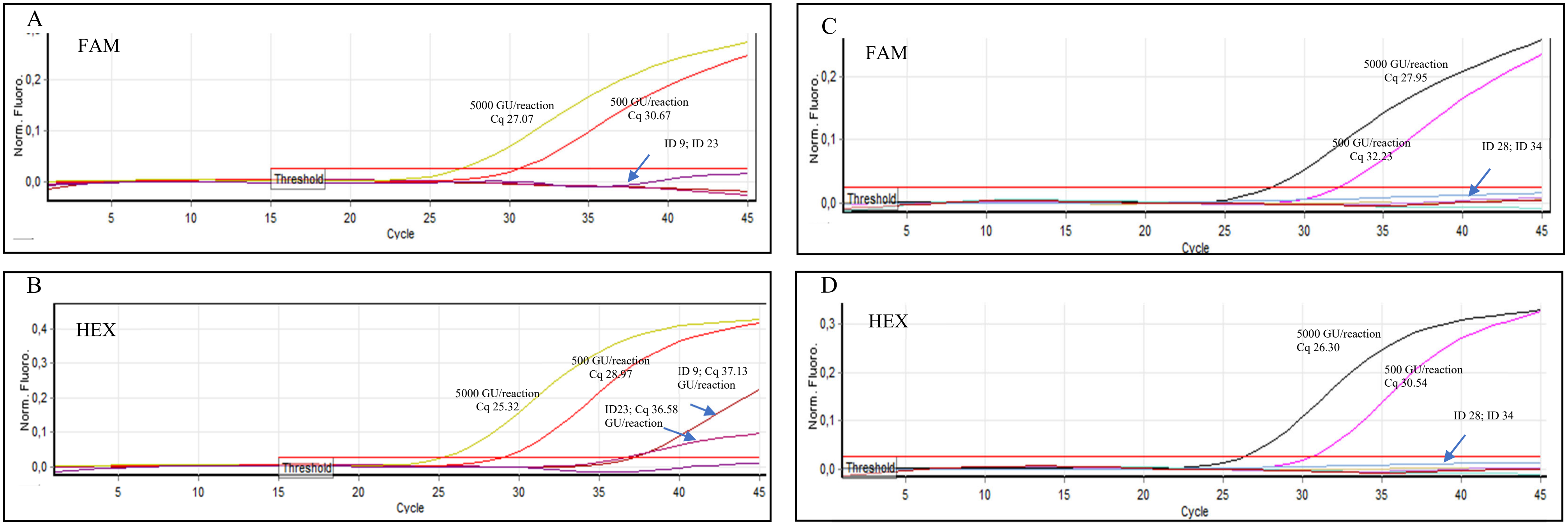

Figure S3. **Rappresentative qPCR amplification curves of low-copy-number discordant samples.** Rappresentative qPCR amplification curves, obtained from a single analytical run of ID9 and ID23 (A-B), ID28 and ID 34 (C-D) samples are shown. FAM for *wzm* gene (A-C) and HEX for *mip* gene (B-D) fluorescence amplitudes, and *Cq* are reported on the x- and y-axes, respectively. The corresponding no-template controls (negative control) are under the threshold. Quantitative results expressed as genomic units (GU) for all these samples are reported in Table S1. The images were captured from the RotorGene Q instrument.

Table S1. Comparison of all results collected by UAT, culture, qPCR and dPCR.

**ID sample**	**UAT**	**Culture**	**qPCR**	**dPCR**	**qPCR (Genomic copies)***		**dPCR (Genomic copies)***		**dPCR (N. of positives)****	
					wzm gene target	mip gene target	wzm gene target	mip gene target	wzm gene target	mip gene target
1	Negative	Negative	Negative	Negative	0	1,59E+00	9,90E-01	9,90E-01	1	1
2	Negative	Negative	Positive	Positive	2,62E+00	8,68E+00	8,10E+00	4,89E+01	8	48
3	Negative	Negative	Positive	Positive	2,86E-01	3,76E+00	9,90E-01	2,35E+01	1	23
4	Negative	Negative	Positive	Positive	3,17E-01	2,56E+00	2,07E+00	3,17E+01	2	31
5	Positive	Negative	Positive	Positive	3,34E-01	2,96E+00	1,35E+00	1,67E+01	1	16
6	Positive	Negative	Negative	Positive	5,46E-02	4,04E-01	9,18E+00	6,63E+01	9	65
7	Negative	Negative	Positive	Positive	2,13E-01	4,36E+00	7,16E+00	3,42E+01	7	33,5
8	Positive	Negative	Positive	Positive	1,14E+01	1,32E+01	3,57E+01	4,68E+01	35	45,7
**9**	**Negative**	**Negative**	**Negative**	**Positive**	**5,28E-01**	**1,26E+00**	**1,02E+00**	**1,36E+01**	**1**	**13,33**
10	Negative	Negative	Positive	Positive	0,00E+00	2,28E+00	7,11E+00	3,92E+01	7	38,5
11	Positive	Negative	Negative	Positive	1,79E-01	1,07E+00	2,07E+00	4,14E+00	2	4
12	Negative	Negative	Negative	Negative	0,00E+00	2,48E-01	1,53E+00	0,00E+00	1,5	0
13	Positive	Positive	Positive	Positive	2,29E+03	2,25E+03	8,22E+03	1,03E+04	6602,5	7930,5
14	Positive	Positive	Positive	Positive	7,80E+00	8,09E+00	3,43E+01	2,77E+01	33,5	27
15	Negative	Positive	Positive	Positive	2,30E+02	2,50E+02	2,28E+02	2,74+02	229	275
16	Negative	Negative	Positive	Positive	1,09E+00	1,31E+01	3,06E+00	7,65E+00	3	7,5
17	Negative	Negative	Positive	Positive	9,44E-01	5,15E+01	1,53E+00	4,23E+01	1,5	41,5
18	Positive	Positive	Positive	Positive	3,78E+00	2,48E+01	6,12E+00	1,49E+01	6	14,5
19	Negative	Negative	Positive	Positive	4,77E-01	2,13E+00	1,44E+01	1,80E+01	13,7	18
20	Negative	Positive	Positive	Positive	5,04E+03	6,67E+03	4,92E+03	6,31E+03	4295,5	5340,5
21	Negative	Negative	Positive	Positive	1,57E+02	2,28E+02	2,52E+02	2,92E+02	246	285
22	Positive	Positive	Positive	Positive	5,36E+03	5,30E+03	5,31E+03	6,16E+03	4541	5153,33
**23**	**Negative**	**Negative**	**Negative**	**Positive**	**2,17E-01**	**1,74E+00**	**0,00E+00**	**6,12E+00**	**0**	**6**

24	Positive	Positive	Positive	Positive	3,56E+03	2,74E+03	5,52E+03	6,28E+03	4283	4720
25	Negative	Negative	Negative	Negative	8,90E-02	9,04E-01	0,00E+00	9,90E-01	0	1
26	Positive	Positive	Positive	Positive	0,00E+00	3,94E+00	2,34E+01	3,51E+01	23	34,5
27	Positive	Positive	Positive	Positive	2,03E+03	1,96E+03	1,77E+03	1,90E+03	1647	1766
**28**	**Negative**	**Negative**	**Negative**	**Positive**	**0,00E+00**	**8,77E-01**	**3,06E+00**	**3,06E+00**	**3**	**3**
29	Negative	Positive	Negative	Positive	0,00E+00	9,46E-01	3,67E+00	1,03E+01	3,67	10,33
30	Positive	Positive	Positive	Positive	3,48E+02	3,30E+02	3,74E+02	4,38E+02	375	439
31	Positive	Positive	Positive	Positive	6,80E+07	1,33E+08	1,10E+05	1,17E+05	20097,5	20149,5
32	Positive	Positive	Positive	Positive	2,43E+00	3,86E+00	1,22E+01	2,09E+01	12	20,5
33	Negative	Negative	Positive	Negative	0,00E+00	2,50E+00	0,00E+00	9,90E-01	0	1
**34**	**Negative**	**Negative**	**Negative**	**Positive**	**9,20E-01**	**1,75E-01**	**9,90E-01**	**3,06E+00**	**1**	**3**
35	Negative	Negative	Positive	Positive	0,00E+00	1,99E+00	9,90E-01	8,19E+00	1	8
36	Positive	Negative	Negative	Negative	0,00E+00	1,29E+00	2,07E+00	2,07E+00	2	2
37	Negative	Positive	Positive	Positive	0,00E+00	2,42E+05	2,07E+00	1,43E+05	2	20442
38	Negative	Negative	Positive	Positive	6,96E+01	5,43E+01	7,24E+01	1,11E+02	71	109
39	Positive	Positive	Positive	Positive	1,68E+01	3,36E+01	2,04E+01	2,85E+01	20	28
40	Negative	Positive	Positive	Positive	0,00E+00	7,75E+01	0,00E+00	1,20E+02	0	120
41	Positive	Positive	Positive	Positive	5,30E+02	2,25E+04	6,63E+02	7,22E+04	636	19665
42	Positive	Negative	Negative	Positive	5,83E-01	2,29E+00	0,00E+00	3,06E+00	0	3

*Data are the average of two independent experiments. ** Number of positive partitions (target copies).
Samples qPCR negative / dPCR positive are in Bold.
